# P-397. Rapid Prediction of Genetic Relatedness of *Escherichia coli* Direct from Critical Care Samples Using Metagenomic Sequencing Paired with Neighbour Typing

**DOI:** 10.1093/ofid/ofae631.598

**Published:** 2025-01-29

**Authors:** Amanda C Carroll, Leanne Mortimer, Hiren Ghosh, Sandra Reuter, Hajo Grundmann, Karel Brinda, William P Hanage, Angel Li, Andrew Purssell, Bryan Coburn, Ashley Rooney, Shola Able-Thomas, Martin Antonio, Derek MacFadden, Allison McGeer

**Affiliations:** The Ottawa Hospital Research Institute, Ottawa, Ontario, Canada; University of Ottawa, Ottawa, Ontario, Canada; University of Freiburg, Medical Center University of Freiburg, Baden-Wurttemberg, Germany; University of Freiburg, Medical Center University of Freiburg, Baden-Wurttemberg, Germany; University of Freiburg, Medical Center University of Freiburg, Baden-Wurttemberg, Germany; Inria, Le Chesnay-Rocquencourt, Ile-de-France, France; Harvard T. H. Chan School of Public Health, Boston, Massachusetts; Sinai Health System, Toronto, Ontario, Canada; Ottawa Hospital Research Institute, Toronto, Ontario, Canada; University Health Network, Toronto, Ontario, Canada; University of Zurich, Zurich, Zurich, Switzerland; Medical Research Council Unit The Gambia at LSHTM, Fajara, Banjul, Gambia, The; Medical Research Council Unit The Gambia at LSHTM, Fajara, Banjul, Gambia, The; The Ottawa Hospital Research Institute, Ottawa, Ontario, Canada; Mt. Sinai Hospital, Toronto, Ontario, Canada

## Abstract

**Background:**

Determining relatedness of bacterial pathogens within hospitals is important to rule out transmission events, but current approaches are typically slow and/or resource intensive. We sought to assess the utility of rapid long-read sequencing with nearest neighbour-typing for identifying relatedness direct from specimens.

**Figure:**
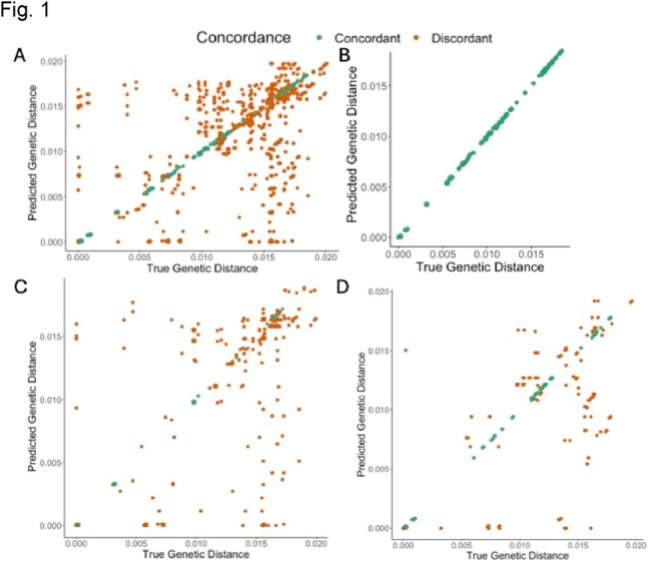

**Figure 1.** Plot of true and predicted genetic distances for E. coli. (a) All comparisons. (B) Only concordant comparisons. (C) All comparisons with LS<0.5. (D) All comparisons with LS>0.5. Green data points are predictions based on concordant calls, and orange data points are predictions based on discordant calls.

**Methods:**

We sequenced remnant urine (n=71) and respiratory (n=7) samples with confirmed *Escherichia coli* (n=78) using ONT MinION, from critical care units at two large tertiary care institutions in Ontario, Canada. The resistance RASE method was used to predict the nearest neighbour for all samples. Using the nearest neighbours and the known reference database phylogeny, we predicted genetic (pairwise) distances (percentage). We then compared the predicted genetic distances with the true genetic distances. Plots visualizing the true and predicted genetic distances were generated and linear models performed. Genetic distance predictions were categorized as: (1) concordant (matching predicted and true sequence types) vs. discordant; and (2) high confidence in prediction (lineage score [LS] >0.5) vs. low confidence in prediction (LS < 0.5).

**Results:**

For all specimens, predicted genetic distances demonstrated a modest but significant correlation (R^2^=0.52, p< 0.001) with true genetic distances (Fig 1A). Predictions of genetic distance were most accurate when the predicted sequence type matched the true sequence type (R^2^=0.99, p< 0.001) (Fig 1B). Using the lineage score improved the accuracy of genetic distance predictions (R^2^=0.744, p< 0.001) (Fig 1C-D). Using a genetic distance cut-off of 0.002 to denote unrelated (non-transmitted) events, as determined by the frequency distribution of genetic distances, a lineage score informed approach could identify genetically unrelated *E. coli* with a specificity of 0.99, sensitivity of 0.96, and positive predictive value of 0.99.

**Conclusion:**

Rapid predictions of genetic-relatedness can be made directly from specimens using long-read sequencing paired with neighbour-typing. We demonstrate that in high confidence calls, the potential for related transmission between infections can be effectively ruled out.

**Disclosures:**

**Allison McGeer, MD**, AstraZeneca: Honoraria|GSK: Honoraria|Merck: Honoraria|Moderna: Honoraria|Novavax: Honoraria|Pfizer: Grant/Research Support|Pfizer: Honoraria|Roche: Honoraria|Seqirus: Grant/Research Support|Seqirus: Honoraria

